# Pupil size tracks attentional breadth in the Navon task

**DOI:** 10.1177/03010066251345778

**Published:** 2025-06-17

**Authors:** Martin Teunisse, Damian Koevoet, Ydo Baarda, Chris L. E. Paffen, Stefan Van der Stigchel, Christoph Strauch

**Affiliations:** Experimental Psychology, Helmholtz Institute, 8125 Utrecht University, The Netherlands; Experimental Psychology, Helmholtz Institute, 8125 Utrecht University, The Netherlands; Experimental Psychology, Helmholtz Institute, 8125 Utrecht University, The Netherlands; Experimental Psychology, Helmholtz Institute, 8125 Utrecht University, The Netherlands; Experimental Psychology, Helmholtz Institute, 8125 Utrecht University, The Netherlands; Experimental Psychology, Helmholtz Institute, 8125 Utrecht University, The Netherlands

**Keywords:** spotlight of attention, attentional breadth, visual attention, local, global, effort, navon

## Abstract

Processing limitations necessitate the selection and prioritization of parts of the visual input—that is visual attention. Visual attention cannot just shift in space, but also changes in size, so-called attentional breadth. A common paradigm to assess attentional breadth is the Navon task wherein participants are instructed to attend global or local features in ambiguous figures. Differences in response times and accuracy then allow inferences about attentional breadth. Here we tested an alternative, overt-behavior free marker of attentional breadth in the Navon task: pupil size changes. Participants were asked to report the parity of either the global or the local number making up an adjusted Navon stimulus. Global and local numbers differed in luminance. We found no differences in pupil size when either a bright or dark feature was attended. However, we did find a larger pupil size when the global compared with when the local number was attended. This effect could be attributed to multiple factors. First, as accuracy was lower when reporting global compared with local features, task difficulty likely affected pupil size. Second, the observed effect possibly reflects higher effort necessary for a wide compared with a narrow attentional breadth—in our specific task layout. Third, we speculate that attentional breadth may effort-independently contribute to this difference in pupil size. Future work could tease apart these factors by changing task layout and stimulus sizes. Together, our data show that pupil size may serve as a physiological marker of attentional breadth in the Navon task.

## Introduction

Only a fraction of the visual input reaching the retina can be processed at any given time. These processing limitations necessitate the selection and prioritization of certain visual information for further processing (i.e. visual attention (Broadbent, 1965; Carrasco, 2011; Petersen & Posner, 2012; Posner, 1980). Similar to a stage setting in a theater, visual attention can be conceptualized as a spotlight (Posner, 1980) that is directed across a scene. This model is extended by the zoom-lens theory of spatial attention (Eriksen & St James, 1986), which postulates that the attentional spotlight has a variable size—referred to as attentional breadth—and intensity. Attentional breadth influences whether perception prioritizes global (the “forest”) or local (the “trees”) features, even when both interpretations are equally plausible.

**Figure 1.**
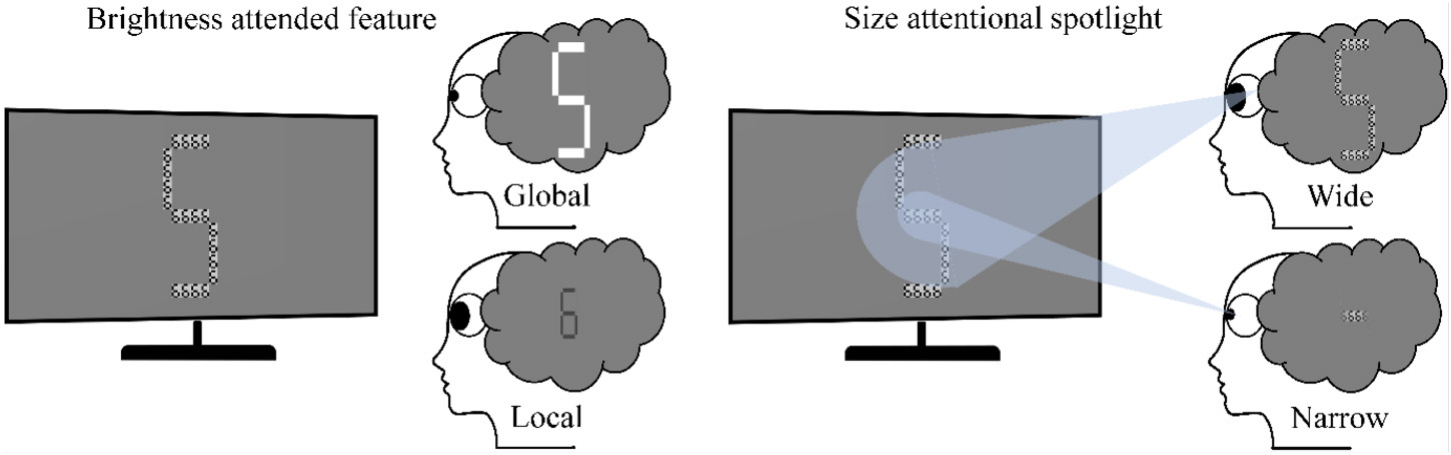
Example stimulus material and hypotheses. Two potential mechanisms to track attentional breadth in the Navon task were explored. Left: We expected the brightness of the attended number to affect the pupil light response more than the brightness of the unattended number. Right: We expected attention to the global number to be associated with relatively larger pupil size than attention to the local number. This could be due to higher effort needed to report the global feature or to optimize the pupil for peripheral vision

Attentional breadth has, perhaps most prominently, been investigated using the Navon task (Navon, 1977). In the Navon task, a global shape, such as a letter or digit, is constructed of many small local shapes (See Figure 1). Inferences about the attentional breadth to either global (overall shape) or local (the individual components making up the overall shape) are drawn from response times or accuracy differences when instructed to report either feature. And indeed, behavioral measures of the Navon task have delivered a wealth of findings on differences in attentional breadth across differing goals or attentional states (Gasper & Clore, 2002; Kolnes et al., 2022). Furthermore, inter-individual differences, such as age (Lux et al., 2008, e.g.), clinical alterations (de Fockert & Cooper, 2014; Muth et al., 2014; Richard & Lajiness-O’Neill, 2015; Ten Brink et al., 2024), or even more indirect and abstract concepts such as culture (McKone et al., 2010) are associated with different behavioral outcomes of the Navon task. Note, however, that there is not one single version of the Navon task. Rather, several different versions of task design and specific instructions exist. For instance, participants can be instructed to report a target letter or number that is present at a local or global level or completely absent in so-called undirected Navon tasks. Alternatively, participants can be instructed to report the global or local feature in so-called directed Navon tasks, as in the present study (see Goodhew, 2020, for a review).

While the Navon task is frequently used to measure attentional breadth, its limited reliability (Dale & Arnell, 2013; Hedge et al., 2018) and reliance on overt manual responses that might affect attentional breadth itself and only provide snapshot insights pose limitations. We here aimed to test a physiological and objective measure of attentional breadth within the Navon task: pupil size changes. Although EEG and fMRI studies have successfully tracked attentional breadth (Feldmann-Wüstefeld & Awh, 2020; Sasaki et al., 2001), they rely on expensive and complicated methods and analyses. These neuroimaging approaches may therefore not be feasible when addressing specific questions such as when investigating inter-individual differences. We adopted pupillometry, an affordable, continuous, and unobtrusive measure, to index attentional breadth in an adjusted version of the Navon task. What specific properties of pupil size changes could allow pupillometry to be an objective measure of attentional breadth?

First and foremost, the pupil responds to changes in brightness in the so-called pupil light response (*PLR*), with constrictions to increases in brightness and dilations to decreases in brightness. Interestingly, the PLR is not purely reflexive but is modulated by the degree of attention to brightness and higher-level interpretations of brightness (Binda & Gamlin, 2017; Binda et al., 2013a; Laeng & Endestad, 2012; Laeng & Sulutvedt, 2014; Mathôt & Van der Stigchel, 2015; Naber & Nakayama, 2013; Strauch, 2024; Strauch et al., 2022). At constant overall brightness and fixation, covertly attending to relatively brighter/darker stimuli constricts/dilates the pupil, respectively (Binda et al., 2013b; Haab, 1886; Mathôt et al., 2013; Naber et al., 2011; Naber & Nakayama, 2013; Strauch, 2024; Strauch et al., 2022). Interestingly, Tkacz-Domb and Yeshurun (2018) showed that attentional modulations of the PLR may be used to track attentional breadth. In their study, dark/bright probes were presented around an upcoming target location in space. Brightness-tagged probes only modulated pupil responses only if they were presented close to the attended target location, suggesting that attentional breadth may be tracked through the PLR (Tkacz-Domb & Yeshurun, 2018). Together, these findings demonstrate that attentional modulations of the PLR can track what is covertly attended, at least when different luminances are clearly separated in space. Here, our goal was to capitalize on this principle in the Navon task by tagging the global and the local features with opposing luminances (e.g., a white local stimulus goes in hand with a black shaded background forming the global stimulus, see Figure 1 for an example). Assuming that these differences would be sufficient to elicit attentional modulations of the PLR, this would allow us to infer the attended feature. Our reasoning was therefore the following: Attending the bright feature should go in hand with smaller pupil size, attending the darker feature should go in hand with larger pupil size (see Figure 1 left for an illustration of the hypothesized effect and mechanism).

Second, a larger attentional breadth has been associated with larger pupil sizes, also in situations with equiluminant stimuli (Brocher et al., 2018; Daniels et al., 2012; Klatt et al., 2021; Kolnes et al., 2024; Vilotijevic & Mathôt, 2023a, 2023b). Since the pupil dilates strongly with increasing mental effort (Beatty, 1982; Bumke, 1911; Kahneman, 1973; Koevoet et al., 2024b; Laeng & Endestad, 2012; Sirois & Brisson, 2014; Strauch, 2024; Strauch et al., 2022; van der Wel & van Steenbergen, 2018), larger attentional breadth could show in increased pupil sizes due to higher *effort* associated with large compared with small attentional breadths (Brocher et al., 2018; Klatt et al., 2021). Hereby, effort refers to the subjective investment of cognitive resources by the participant, for instance, to meet the task demands. Effort relates to task difficulty, but task difficulty is not the only factor affecting effort and the link between difficulty and effort is inherently non-linear. Increasing task difficulty shows in more effort exertion at first (and pupillary expansion, Bumke, 1911; Kahneman, 1973; van der Wel & van Steenbergen, 2018), but when capacity is exceeded, participants disengage and both effort exertion and pupil size are reduced (i.e. giving up, Granholm et al., 1996). Because task difficulty is not the only factor affecting effort exertion, any other effortful factor may still affect pupil size at constant task difficulty. As the only study to date using pupillometry in the context of a Navon task, DiCriscio et al. (2018) examined pupil responses associated with the selection of global versus local features. They found larger pupil sizes when participants were instructed to report the global rather than the local feature. Accuracy was at ceiling across conditions (DiCriscio et al., 2018), but participants responded faster in the global than in the local condition, suggesting higher difficulty for local processing. This finding challenges a pure effort-based explanation of the larger pupil sizes observed in the global condition (DiCriscio et al., 2018). A complication arises from a potential confound of motor preparation differences between the global and local conditions. Since pupil dilation was larger in the global condition, where responses were also faster, and motor responses are known to elicit sizable pupil dilations (Bumke, 1911; Richer & Beatty, 1985; Strauch et al., 2020), it remains unclear whether the observed effect reflects wider attentional breadth or simply a consequence of pupil dilation effects caused by motor preparation/execution. Besides reflecting effort, larger pupil sizes have indeed been associated with increased detection performances at the periphery at the cost of foveal acuity (Eberhardt et al., 2022; Mathôt & Ivanov, 2019), also when controlling for task difficulty (Ruuskanen et al., 2024; Vilotijevic & Mathôt, 2023a). In other words, larger pupil sizes that go hand in hand with larger attentional breadth are optically more optimal for the perception of global features (Vilotijevic & Mathôt, 2023b). Together, we therefore expected pupil size to scale with the size of attentional breadth: Attention to global features should go in hand with a larger pupil size, whilst attention to local features should go in hand with a smaller pupil size, respectively (see Figure 1 right for an illustration of the hypothesized effect and mechanism).

We therefore tested whether pupil responses can serve as an objective marker of attentional breadth in an adjusted Navon task in two ways: First, we tested whether we can infer attentional breadth by modulations of the PLR that correspond to the brightness of the attended feature, respectively. Second, we tested whether larger pupil sizes are indicative of attending global or local features through effort-linked pupil size changes and/or attentional breadth affecting pupil size to optimize vision even irrespective of effort.

## Methods

All data and analysis scripts are available here: https://osf.io/5dqcn/.

### Participants

A total of 30 participants with normal or corrected-to-normal vision were tested after providing informed consent. Participants were drawn from a general student population at Utrecht University. While detailed demographic information was not collected, the sample is assumed to be representative of the typical characteristics of students at a Dutch university. Sample size was based on a pilot study and sample sizes by Brocher et al. (2018), featuring 32, 23, and 24 participants per experiment. Compensation was given either in the form of course credits or €8/h in cash. The ethics committee of Utrecht University’s Faculty of Social and Behavioral Sciences approved the experiment (22-0580).

### Apparatus

The experiment was conducted in a sound and light-controlled room using an EyeLink 1000+ (SR Research, Mississauga, Ontario, Canada). Stimuli were presented on a 27” 2560 × 1440 LCD monitor with a refresh rate of 100 Hz. Participants were seated and stabilized with a chin- and forehead rest at 67.5 cm from the monitor. Gaze position and pupil size of the left eye were recorded at 500 Hz. Audio cues were played through the speakers of the monitor to indicate the feature the participants had to report. The experiment was implemented using PsychoPy (v2021.2.3 Peirce et al., 2019).

### Material

**Figure 2.**
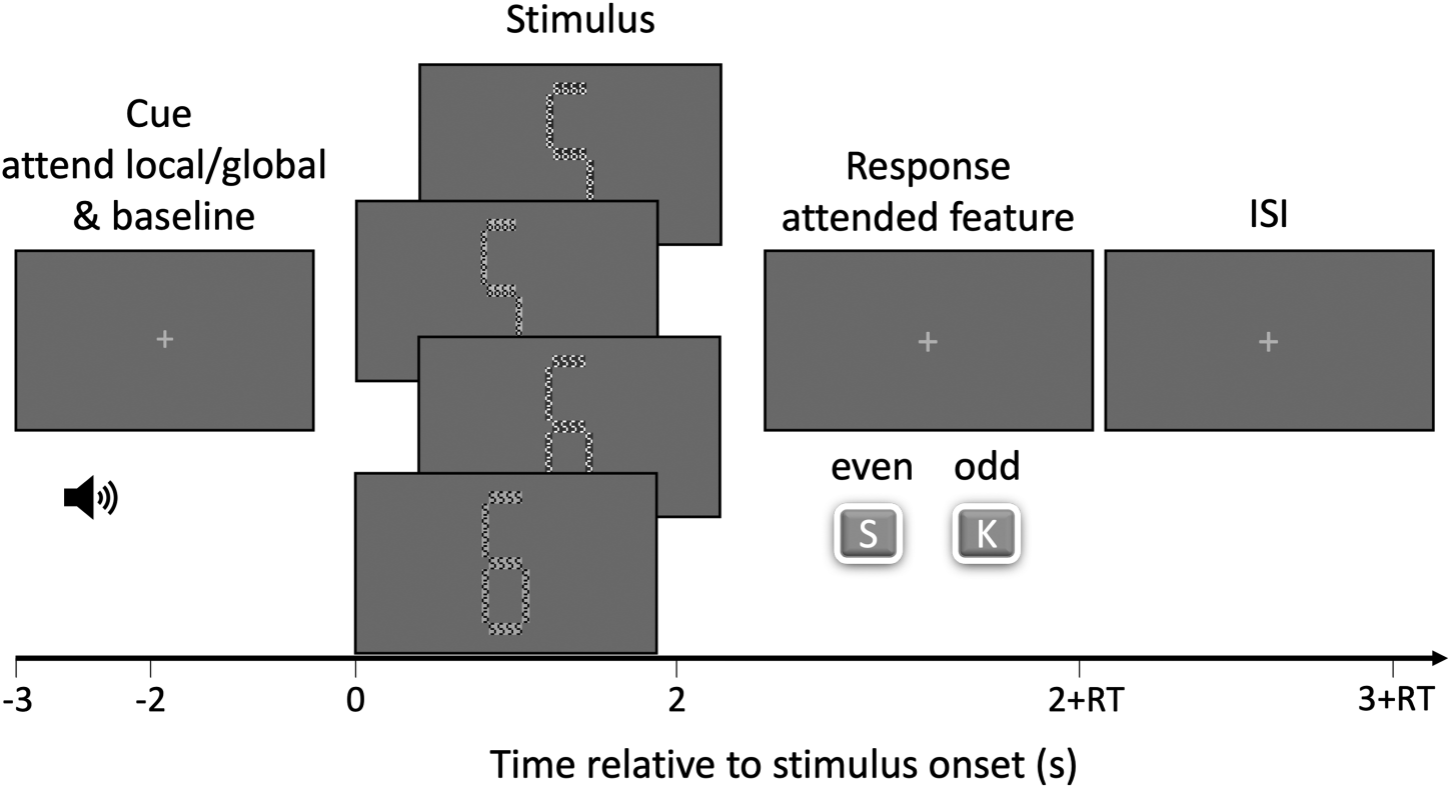
Trial procedure. A 1 s auditory cue with a high or low pitched tone indicated which feature to report (global or local). The next 2 s constituted a baseline period. After this, the stimulus was presented for 2 s. Stimuli always contained one global and local feature that differed in brightness and parity. Participants reported the parity of the attended feature after stimulus offset. A blank inter stimulus interval of 1 s was used.

Stimuli (global number: 4.18° × 12.62°, local number: 0.61° × 1.06°) were presented on an intermediate gray background. Note that stimuli here were particularly large, especially in the vertical dimension. Yet, expanding attentional breadth horizontally to both edges of the much smaller horizontal axis sufficed to report number identity correctly. Stimuli were based on the classic Navon letter stimuli (Navon, 1977), but we instead used digital numbers (2–9, excluding 4 and 7, as a global 1 and 7 do not cover central fixation, 4 to have an equal number of odd and even stimuli) (Figure 2). For each stimulus, either the global or the local number was even, whereas the respective other number was odd. Two brightness combinations were used, one with global numbers being darker than local numbers and vice versa. We independently manipulated the tagged brightness and spotlight size (Figure 1). In other words, bright or dark local features and bright or dark global features occurred equally often. The overall grayness of the stimulus on any given trial always equaled the background color. Each number covered a specific number of pixels. To balance global and local brightnesses out to average gray, digits covering less space were therefore of more extreme brightness. Stimuli were created with Python 3.9 and GIMP 2.10.32. The combination of odd and even, and black and white resulted in 36 unique stimuli.

### Procedure, Design, and Task

Before the experiment, a 5-point calibration and validation of the eye-tracker was conducted. Participants were instructed to report the parity (odd/even) of the cued feature (global or local) of the stimulus. Trials started with an auditory cue tone (1 s) that indicated whether the parity for the global or local number had to be reported (high (440 Hz) or low (294 Hz) tone counterbalanced across participants). Based on the existing work using the Navon task, we assumed that the instruction of attending the local feature would induce a local and therefore small attentional breadth and that attending the global feature would induce a wide attentional breadth. A subsequent 2 s baseline phase featured a single black fixation dot in the center of the screen (radius 0.1°) and was continuously present during the baseline and stimulus phase. Afterwards, the stimulus was presented for 2 s, and participants subsequently reported whether numbers were even (s) or odd (k) to ensure that participants attended the instructed feature. To further ensure close attention to the feature, 10% of trials were catch trials in which the attended number changed to the letter “E” or “F” for 100 ms after 450 ms of stimulus presentation. Participants were instructed to press the “space” key as fast as possible if a change to the letter E occurred, and to continue as usual when a change to the letter F occurred. If the participant missed a change to the letter E, or incorrectly responded to a change to the letter F, a red circle would appear after the participant responded to the original task. Participants completed three blocks of the experiment, with each block comprising all stimuli presented in random order. Consequently, each stimulus combination was presented three times per participant.

To prevent distortions on pupil size, participants needed to keep gaze position central (Mathôt & Vilotijevic, 2023; Strauch et al., 2022). To ensure central gaze position during the task, trials were deemed invalid if gaze position deviated from the center by more than 3.39°visual angle (75 px) for more than 250 ms (to allow for occasional blinks). This precluded pupil foreshortening errors (see Strauch et al., 2022) and ensured that participants changed attentional breadth rather than shifting gaze. Note that results remained qualitatively unchanged when only considering trials within 1°visual angle after drift correction (based on the first 10 ms after stimulus onset). Invalid trials were fed back by displaying “Incorrect gaze” on screen. To ensure valid data for all participants, invalid trials were repeated later in the same block. To reduce the influence of (preparing) the motor response on pupil size, participants were only allowed to respond after the stimulus was off screen.

### Data Preprocessing

Custom python scripts were used to process and analyze the data. Behavioural performance was assessed by computing average accuracies and response times for each condition. Accuracy and response times were analysed using two-way repeated-measures analysis of variance (ANOVA) (2 brightnesses × 2 global/local) in JASP (v0.17).

Pupil analyses were based on previous work (Koevoet et al., 2024; Strauch et al., 2022). Pupil size data were epoched from stimulus onset and blinks were interpolated (Mathôt & Vilotijevic, 2023). We applied subtractive baseline correction using the median pupil size of the first 250 ms. For statistical analyses, linear mixed-effects (LMEs) models were used to analyze pupil size over time (every 20 ms). LMEs were fit to test for the effects of condition (global versus local) and brightness of the to-be attended feature as well as their interaction on pupil size. The model incorporated both condition and brightness in the following formula (Wilkinson Notation): Pupil size ∼ Condition * Brightness + (1 + Condition + Brightness—Participant). We modeled by-participant intercepts and slopes to limit Type 1 errors (Barr, 2013). Model assumptions were checked for the LME fit to the average of timepoints 500–2000 ms; no model violations were found. Note that we did not test model assumptions for each model over time. We set our threshold for significance at α = 0.05 for all analyses. To address the issue of multiple comparisons, we applied the Benjamini-Hochberg false discovery rate (FDR) correction on the *p*-values obtained from the LME analysis over time.

## Results

### Manipulation Check

Prior to our main analyses, we analyzed the performance on the catch trials to assess whether participants were properly following task instructions. To this end, we conducted one-sample *t*-tests to test whether participants scored above chance level (50%) on the catch trials. Although the catch trials were relatively challenging (*M*_Accuracy_ = 56.9%, 95% CI = [54.6–59.2%]), participants performed above chance on the catch trials in the global (*t*(29) = 2.34, *p* = 0.027, Cohen’s *d* = 0.43) and local conditions (*t*(29) = 2.34, *p* = 0.026, Cohen’s *d* = 0.43). This established that participants followed task instructions properly.

### Behavioral Results

**Figure 3.**
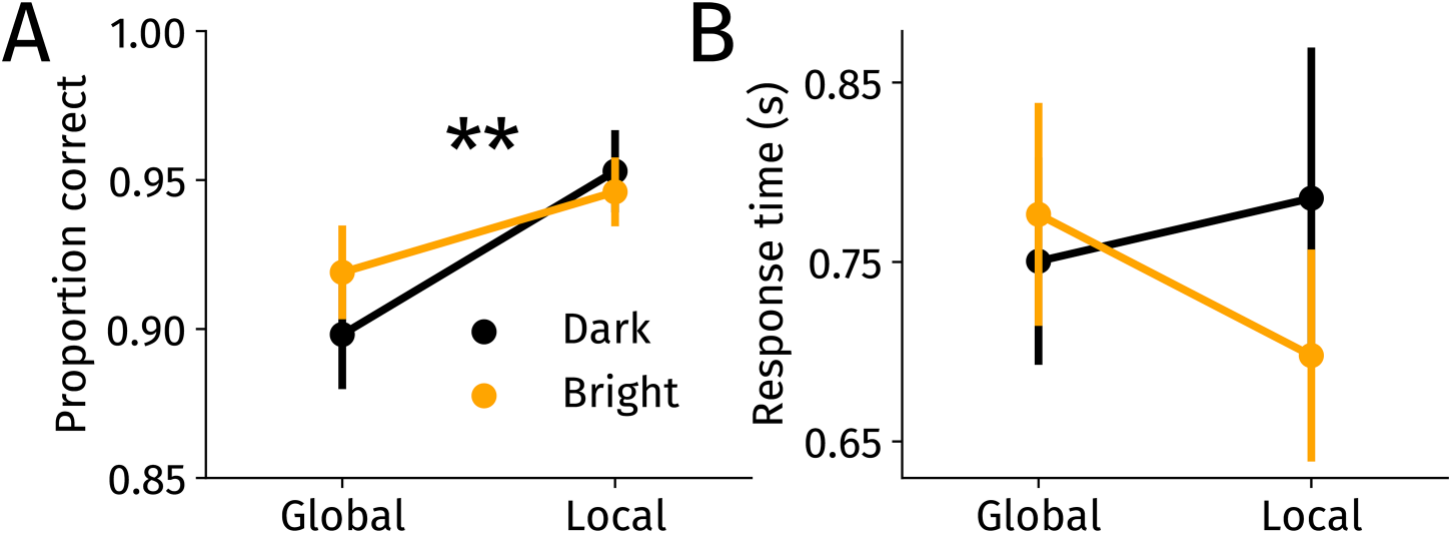
(A) Proportion of correct responses across conditions. (B) Average response times across conditions. Note that response times have to interpreted with caution due to the delayed response. Error bars indicate standard errors of the mean. **p <0.01 .

The Navon task yields both accuracy and response time (Figure 3). Accuracy on the current task may inform about the tendency towards a narrow versus wide attentional breadth, respectively. A two-way repeated-measures ANOVA (2 brightness × 2 global/local) was used to analyze accuracy between conditions (data did not differ from normal distribution). Accuracy was higher in the local compared with the global condition (*F*(1,29) = 11.03, *p* = 0.002, $\eta_{\;p}^{2}$ = 0.28; Figure 3A). Additionally, accuracy did not differ significantly between different brightness levels (*F*(1,29) = 0.38, *p* = 0.54, $\eta_{\;p}^{2}$ = 0.01), and the interaction effect was also not significant (*F*(1,29) = 2.01, *p* = 0.16, $\eta_{\;p}^{2}$ = 0.07). The analysis shows that identifying the parity of a global compared to a local number was more difficult and that there was no reliable effect of brightness on accuracy.

Average response times were analyzed using a two-way repeated-measures ANOVA (as in the accuracy analysis) (Figure 3B). None of the effects were significant, indicating that neither the to-be-attended brightness, nor the to-be-reported feature reliably affected response times (*F*s(1,29) < 2.7, *p*s > 0.10, $\eta_{\;p}^{2}$ < 0.09). However, we note that participants had to withhold their response until stimulus offset. This may have led to diminished effects on response times, and we therefore urge caution when interpreting these null results.

### Pupil Analysis

**Figure 4.**
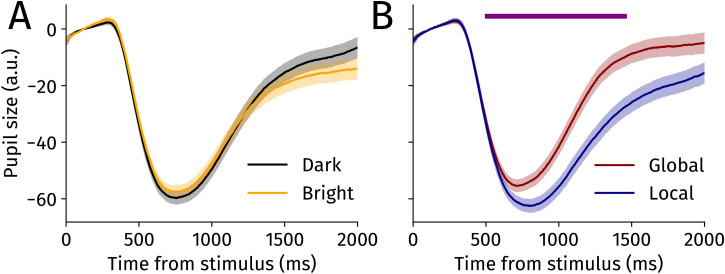
(A) Pupil size for dark versus bright trials over time. (B) Pupil size for global versus local trials over time. The purple line represents whenever *p* < 0.05 after FDR-correction. Shaded error bands reflect standard errors of the mean.

Next, we tested whether pupil size could track attentional breadth. We first investigated whether the different brightnesses of the local and global features revealed which respective feature was attended. We expected a smaller pupil size when attending to the brighter feature (be it global or local) than when attending to the darker feature. Figure 4A depicts pupil traces separately averaged for attention to darker (black) or brighter features (yellow). In contrast to our hypothesis, we observed no significant differences in pupil size between when a bright or a dark number was attended (all FDR-corrected *p*s > 0.90) throughout the entire timecourse. This indicates that the PLR cannot reliably dissociate the breadth of attention in our adjusted Navon task through a brightness-linked mechanism.

We next tested whether attentional breadth modulated pupil size independent of the tagged brightness. The analysis revealed significant differences from approximately 500 ms until 1460 ms after stimulus onset (FDR-corrected *p* < 0.05; horizontal purple line in Figure 4B). These results demonstrate that pupil size was larger in the global condition compared with the local condition. Thus, pupil size tracked attentional breadth as induced by paying attention to global versus local feature independent of the tagged brightness. We observed no significant interaction of to be attended luminance (bright dark) and feature (global/local) throughout the time trace (all FDR-corrected *p*s > 0.16). In a control analysis, we observed no interaction of attended featured with accuracy, showing that the effect of attended feature (global/local) persisted in both correct and incorrectly answered trials. A further control analysis showed that participants with high or low performance on the catch trials showed the same effects on pupil size (Supplemental Material).

Lastly, we wanted to assess the robustness of our findings, as well as obtain an interpretable effect size that can be readily compared with other studies. To this end, we conducted a two-way repeated-measures ANOVA (2 brightness × 2 global/local) on the average pupil size 500–2000 ms after stimulus onset. Complementing our LMEs model analysis over time, we found a larger pupil size in global compared with local trials (*F*(1,29) = 12.64, *p* = 0.001, $\eta_{\;p}^{2}$ = 0.30). Moreover, neither the main effect of brightness, nor the interaction effect between brightness and feature were significant (*F*s(1,29) = 1.26, *p*s > 0.27, $\eta_{\;p}^{2}$ < 0.05). This shows that our results are not dependent on very specific timepoints and are instead robust when averaging across time. Moreover, the effect size of the difference in pupil size between global and local trials was considerable ( $\eta_{\;p}^{2}$ = 0.30). Finally, effects were highly stable within the experiment (see Supplemental Material).

## Discussion

Visual attention helps us to select and amplify parts of the visual input (Carrasco, 2011; Posner, 1980). The focality of attentional selection is malleable (e.g. Feldmann-Wüstefeld & Awh, 2020; Vilotijevic & Mathôt, 2023a), in other words, attentional breadth is not rigid and can vary. A common paradigm to assess attentional breadth is the Navon task, which measures response time and accuracy, while participants are instructed to attend global or local features in ambiguous figures. As an alternative to these overt responses, we here investigated whether attentional breadth can be tracked using pupil size. To this end, we tested whether pupil size can index attentional breadth by manipulating both the brightness of the global and local features in an adjusted Navon stimulus. Participants had to report either the parity of the global or local feature. While the brightness manipulation did not affect pupil size, pupil size tracked attentional breadth differently: Pupil size was larger when instructed to attend globally compared with being instructed to attend locally. Before discussing the implications of this result, we first consider why manipulating the brightness of global and local features failed to affect pupil size for our stimulus.

Our first hypothesis was that the PLR would be modulated by the luminance of the attended feature (i.e., a smaller pupil if the attended feature was bright, a larger pupil when the attended feature was dark). In contrast to our hypothesis, our results showed no significant differences in pupil responses when participants attended either the brighter or darker elements of the stimulus (see Figure 4A). One reason for the absence of the hypothesized effect might be that brightness was task-irrelevant to successfully report the parity of the numbers. However, attentional modulations of the PLR have previously been observed with different brightnesses that are also task-irrelevant (e.g. Binda et al., 2013a, 2013b; Mathôt et al., 2013; Naber & Nakayama, 2013; Ten Brink et al., 2023).

We instead believe that it is most likely that the PLR reflects the overall brightness of an attended location, which would also result in a null-effect in the current study. To illustrate, attending any single “foreground” local digit always has a “background” with the brightness of the global feature. Reversely, attending the brightness of the “background” global feature also includes the opposing “foreground” brightness of the local feature. In both cases, this leads to an average gray brightness within the attentional locus. A modulation of the PLR in this case would have necessitated a separation of the fore- and background brightnesses, which perhaps did not occur or the PLR is simply not sensitive to such a process. One may argue that this interpretation is unlikely based on previous work on modulations of feature-based attention of the PLR (Binda et al., 2014). However, the current situation differs fundamentally from earlier work because here the non-relevant brightness does not interfere with the task, and does not have to be suppressed. Together, we suggest the PLR to reflect the overall brightness of the attended location without reflecting figure-ground separation processes in our task.

Our second hypothesis was that pupil size would reflect attentional breadth irrespective of the brightness of the relevant feature. In line with this hypothesis, we found pupil size to be robustly larger in the global compared with the local condition (Figure 4B). Which underlying mechanism may be responsible for the larger pupil size with larger attentional breadth? As pupil size scales with effort (Beatty, 1982; Bumke, 1911; Kahneman, 1973; Koevoet et al., 2024b; Sirois & Brisson, 2014; Strauch et al., 2022; van der Wel & van Steenbergen, 2018), the observed differences in pupil size might be due to differences in the effort needed to solve the task in a given condition. Lower accuracies in the global compared with the local condition indeed indicate a higher difficulty of the global condition—and therefore it is likely that more effort was required when in global trials. Our finding may also explain why the pupil was previously found larger for a larger attentional breadth in the only other existing study combining the Navon task with pupillometry (DiCriscio et al., 2018), but where a ceiling effect in accuracies did not allow for such conclusions. Pupil size may thus still have captured differences in effort when behavior was already at ceiling performance. This is reminiscent of Brocher et al. (2018) who showed that when shifting attention further into the periphery, effort-related pupil dilations are larger—even at equal task difficulty. However, DiCriscio et al. (2018) who similarly reported larger pupil size for the global condition, found faster response times for global than for local (but note the need for replication with a delayed response). This goes against the notion of larger pupil sizes for the global condition in DiCriscio et al. (2018) to reflect the same differences in task difficulty as found here.

Possibly, the current findings may not be caused by differences in effort, but a larger attentional breadth dilates the pupil to optimize for visual processing irrespective of effort (Vilotijevic & Mathôt, 2023a). Larger pupil sizes for larger attentional breadth might be optimizing vision because larger pupils improve detection in the periphery at the expense of acuity in the center (Eberhardt et al., 2022; Mathôt & Ivanov, 2019; Ruuskanen et al., 2024; Vilotijevic & Mathôt, 2023b). Our working hypothesis is that the here observed larger pupil size in the global condition results from differences in effort in combination with an additional effort-independent effect of attentional breadth on pupil size. Such a larger pupil size under similar difficulty for wider attentional breadth irrespective of differences in effort (Vilotijevic & Mathôt, 2023a) would be in line with larger pupil sizes for global compared with local, despite higher difficulty for local than for global in DiCriscio et al. (2018). Note, however, the potential motor preparation artifact in DiCriscio et al. (2018) outlined in the introduction that could equally underlie larger pupil size for the global condition. Together, this raises the question what is cause and effect: is a pupil size increase a mechanism to enhance peripheral vision, or is it primarily a response to increased effort, with potentially enhanced peripheral detection being a secondary benefit?

Attending to local compared with global features is differently difficult depending on interindividual differences, but also task layout, possibly due to different attentional breadths as a trait (e.g. McKone et al., 2010). Factors such as sparsity/density and relative size of local features (Martin, 1979; Yovel et al., 2001) or stimulus size considerably influence whether local or global conditions are more difficult (Goodhew, 2020; Rezvani et al., 2020), and even the expansion of attentional breadth may be easier across the horizontal meridian than across the vertical (Hüttermann et al., 2014). In contrast to the present findings, a global precedence is often found, that is, global features are typically *easier* to report than local features (Goodhew, 2020). In line with global precedence effects, performing a more difficult working memory dual task seems to selectively impair local but not global performance (Ahmed & De Fockert, 2012; Ahmed & de Fockert, 2012; Caparos & Linnell, 2010; Linnell & Caparos, 2011). However, this global precedence begins to tilt toward a local precedence as stimuli exceed a size of about 6–9 degrees visual angle (Kinchla & Wolfe, 1979) (but see Navon and Norman (1983). Indeed, a substantial effect of stimulus size on global/local precedence is found meta-analytically—with larger stimuli being associated with higher difficulty for global attention (Rezvani et al., 2020). In line with this, it is plausible that our relatively large stimuli elicited a local over a global precedence. The overall effort effect could result from both differences in task difficulty and effort associated with expanding attentional breadth. If the current effects are fully driven by effects of task difficulty and not the effort of expanding attentional breadth, increasing the difficulty for the local feature should flip the pupil effect (i.e., pupil size being larger for local instead of global)—although this seems unlikely given the combination of our and DiCriscio et al. (2018)’s results. Future work should aim to equate task difficulty using staircase procedures (e.g. Vilotijevic & Mathôt, 2023a). At similar difficulty, remaining pupil size differences should arguably not be due to differences in task difficulty, and a relatively pure assessment of the effort associated with expanding or shrinking attentional breadth will be revealed.

We see considerable potential of studying differences in effort associated with different attentional breadths. Just like for expanding or shrinking attentional breadth, shifting the locus of attention is associated with differences in effort that can be measured with pupil dilation. As such differences in effort have been identified across types of attentional shifts (overt/covert) and eye-movement directions (Koevoet et al., 2023, 2025). These differences are in turn highly predictive of eye-movement preferences, with participants preferring affordable (i.e. relatively less effortful) over costly eye movements (i.e. relatively more effortful (i.e. relatively more effortful; Koevoet et al., 2025). In line with this work, attention-inherent cost has been repeatedly suggested as a key driver of visual attention (e.g. Burlingham et al., 2024; Diamond et al., 2017; Hoppe & Rothkopf, 2016; Kadner et al., 2022; Shadmehr & Ahmed, 2020; Thomas et al., 2022). Possibly, similar cost-optimality principles apply to how attention is deployed (Kahneman, 1973; Koevoet et al., 2024, 2025), including its breadth. Following this logic, attentional breadth should be adjusted flexibly under the consideration of the current task and costs. For example, if expanding attentional breadth is costly, one will only expand it if it benefits task performance enough, otherwise a more focal attentional breadth should be preferred. Changes to the costs across states and individuals could therefore hold the key to understanding *why* differences across factors as diverse as attentional state, age, clinical symptomatic, or even culture are found (de Fockert & Cooper, 2014; Lux et al., 2008; McKone et al., 2010; Muth et al., 2014; Richard & Lajiness-O’Neill, 2015; Ten Brink et al., 2024). Pupil size can potentially provide the physiological marker of effort to test this idea further. Connecting the previous two paragraphs, cost-optimality may provide an elegant explanation for why global precedence diminishes, and even reverses, into local precedence when stimuli exceed a certain size (Kinchla & Wolfe, 1979; Rezvani et al., 2020). Either attentional breadth cannot expand beyond a given size, leading to the need to shift a narrow spotlight to integrate global information (see Goodhew, 2020), or it may simply be less cost-optimal to further expand attentional breadth relative to shifting the spotlight across space repeatedly. In line with our prior finding of covert attention shifts being effortful as well (Koevoet et al., 2023), it might then be the need for several shifts to happen that make attention to a larger area effortful. As we previously found spatial attention to follow an effort-minimization principle (i.e., low-effort shifts are performed preferentially; Koevoet et al., 2025), the tipping point from global to local precedence might be the consequence of such an optimization process. This possibility would solve the seeming paradox and inconsistencies around the global precedence effect—cost-optimality of attentional breadth relative to the task has precedence. As pupil size may capture the effort associated with attentional breadth and shifting attention, we see considerable potential to empirically solve this debate.

We here attempted to use pupil size as an objective physiological marker of attentional breadth in an adjusted version of the Navon task. Although we were unsuccessful in leveraging attentional modulations of the PLR to infer attentional breadth, we did find larger pupil sizes for the attend global compared with the attend local instruction. These differences in pupil size, most likely driven by differences in effort, but possibly also reflecting effort-independent due to differences in attentional breadth, offer an objective marker of attentional breadth in the Navon task.

## Supplemental Material

sj-pdf-1-pec-10.1177_03010066251345778 - Supplemental material for Pupil size tracks attentional breadth in the Navon task Supplemental material, sj-pdf-1-pec-10.1177_03010066251345778 for Pupil size tracks attentional breadth in the Navon task by Martin Teunisse, Damian Koevoet, Ydo Baarda, Chris L. E. Paffen, Stefan Van der Stigchel and Christoph Strauch in Perception
