# Supplementary material for: Pupil size tracks attentional breadth in the Navon task
Source: Perception. 2025 Jun 17;54(10):753–67. doi: 10.1177/03010066251345778 (PMC12417611; doi:10.1177/03010066251345778)
Supplement: sj-pdf-1-pec-10.1177_03010066251345778 - Supplemental material for Pupil size tracks attentional breadth in the Navon task [file sj-pdf-1-pec-10.1177_03010066251345778.pdf]

1 Supplementary materials

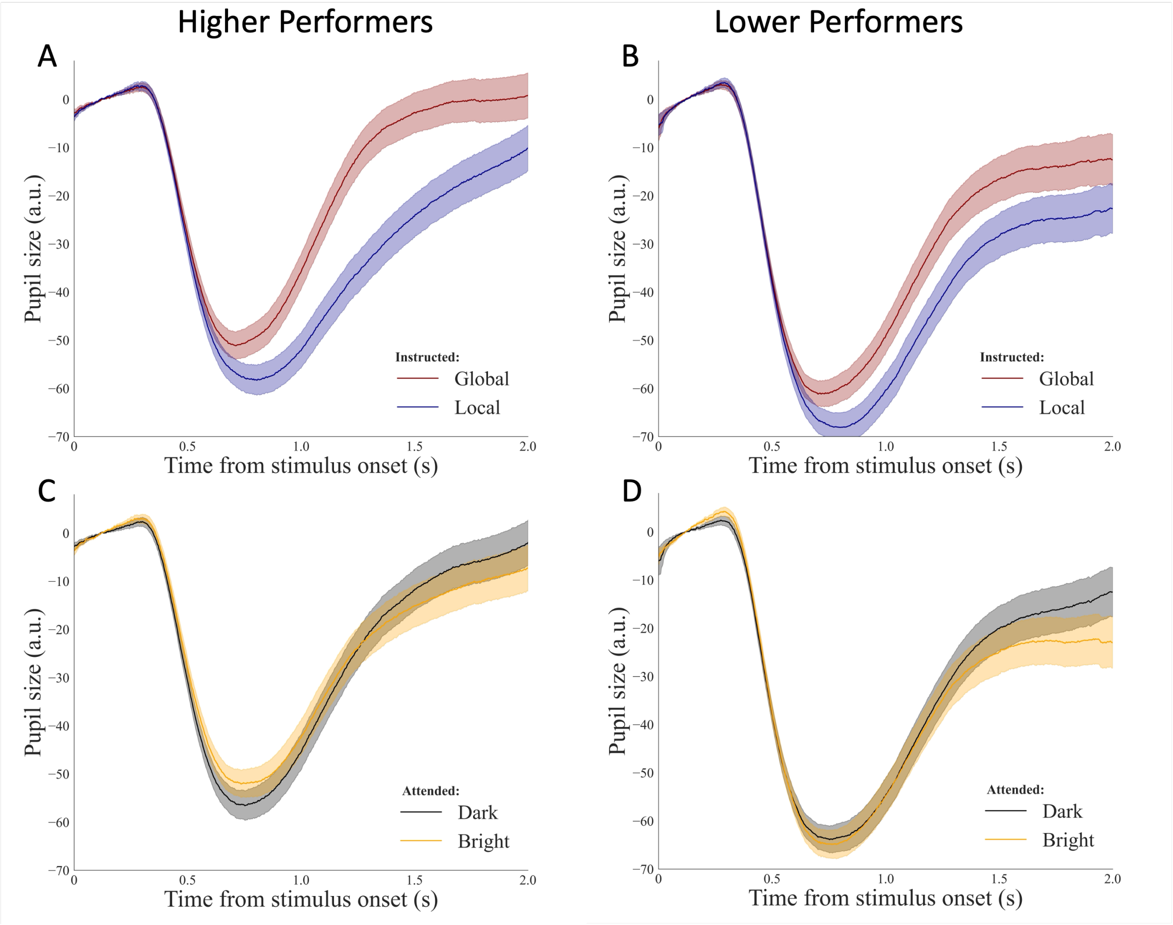

Figure 5: Pupil size analysis based on a median split of participants on the catch trial performance. Low-performers being under the median and high- performers over the median
